# Prostaglandin I2 inhibits IL-33-induced IL-5 and IL-13 production by human type 2 innate lymphoid cells

**DOI:** 10.1186/1939-4551-8-S1-A81

**Published:** 2015-04-08

**Authors:** Weisong Zhou, Kasia Goleniewska, Dawn Newcomb, Jian Zhang, Shinji Toki, R Stokes Peebles

**Affiliations:** 1 Vanderbilt University, USA

## Background

Group 2 innate lymphoid cells (ILC2) are characterized by their expression of cytokines including IL-5 and IL-13 in response to IL-33. ILC2 are critical in mediating influenza virus-induced airway hypersensitivity and are associated with allergic inflammation. However, the factors regulating human ILC2 (hILC2) cytokine responses are not fully defined. In this study, we tested the hypothesis that prostaglandin I_2_ (PGI_2_), a lipid product formed in the cyclooxygenase (COX) pathway of arachidonic acid metabolism, suppresses IL-33-induced cytokine responses by hILC2.

## Methods

hILC2 (Lin-CD25+CD127+ cells) were purified by flow cytometric cell sorting from peripheral blood mononuclear cells and stimulated with IL-33 and IL-2 in the presence of the PGI_2_ analog cicaprost or vehicle.

## Results

We found that hILC2 expressed the IL-33 receptor and the PGI_2_ receptor IP. Treatment of the cells with cicaprost significantly decreased IL-5 and IL-13 production, and the inhibition was associated with lower levels of mRNA expression of the transcription factors involved in the production of these cytokines and ILC2 development including gata3, gfi-1, Ror-α and Id2. cAMP-elevating reagents such as db-cAMP and PGE_2_ had a similar inhibitory effect on IL-5 and IL-13 production by hILC2.

## Conclusions

These data indicate that PGI_2_ inhibits hILC2 cytokine secretion and suggest that use of COX inhibiting drugs may increase the risk of developing allergic diseases by augmenting ILC2 cytokine responses.

